# *NFKB1*-94insertion/deletion ATTG polymorphism and cancer risk: Evidence from 50 case-control studies

**DOI:** 10.18632/oncotarget.14190

**Published:** 2016-12-26

**Authors:** Wen Fu, Zhen-Jian Zhuo, Yung-Chang Chen, Jinhong Zhu, Zhang Zhao, Wei Jia, Jin-Hua Hu, Kai Fu, Shi-Bo Zhu, Jing He, Guo-Chang Liu

**Affiliations:** ^1^ Department of Pediatric Urology, Department of Pediatric Surgery, Guangzhou Institute of Pediatrics, Guangzhou Women and Children's Medical Center, Guangzhou Medical University, Guangzhou 510623, Guangdong, China; ^2^ School of Chinese Medicine, Faculty of Medicine, The Chinese University of Hong Kong, Hong Kong 999077, China; ^3^ Department of Gastroenterology, The First People's Hospital of Foshan (Affiliated Foshan Hospital of Sun Yat-sen University), Foshan 528000, Guangdong, China; ^4^ Molecular Epidemiology Laboratory and Department of Laboratory Medicine, Harbin Medical University Cancer Hospital, Harbin 150040, Heilongjiang, China

**Keywords:** *NFKB1*-94ins/delATTG, polymorphism, cancer risk, meta-analysis

## Abstract

Nuclear factor-kappa B1 (NF-κB1) is a pleiotropic transcription factor and key contributor to tumorigenesis in many types of cancer. Numerous studies have addressed the association of a functional insertion (I)/deletion (D) polymorphism (-94ins/delATTG, rs28362491) in the promoter region of *NFKB1* gene with the risk of various types of cancer; however, their conclusions have been inconsistent. We therefore conducted a meta-analysis to reevaluate this association. PubMed, EMBASE, China National Knowledge infrastructure (CNKI), and WANFANG databases were searched through July 2016 to retrieve relevant studies. After careful assessment, 50 case-control studies, comprising 18,299 cases and 23,484 controls were selected. Crude odds ratios (ORs) and 95% confidence intervals (CIs) were used to determine the strength of the association. The *NFKB1* -94ins/delATTG polymorphism was associated with a decreased risk of overall cancer in the homozygote model (DD vs. II): OR = 0.75, 95% CI = 0.64-0.87); heterozygote model (ID vs. II): OR = 0.91, 95% CI = 0.83-0.99; recessive model (DD vs. ID/II): OR = 0.81, 95% CI = 0.71-0.91; dominant model (ID/DD vs. II): OR = 0.86, 95% CI = 0.78-0.95; and allele contrast model (D vs. I): OR = 0.88, 95% CI = 0.81-0.95). Subgroup and stratified analyses revealed decreased risks for lung cancer, nasopharyngeal carcinoma, prostate cancer, ovarian cancer, and oral squamous cell carcinoma, and this association held true also for Asians (especially Chinese subjects) in hospital-based studies, and in studies with quality scores less than nine. Well-designed, large-scale case-control studies are needed to confirm these results.

## INTRODUCTION

Cancer is a substantial public health burden, with an estimated 1.7 million new cancer cases and 0.6 million cancer-related deaths in the United States in 2016 [1]. Although the etiology of carcinogenesis has not yet been fully elucidated, many lines of evidence suggest that cancer is a multifactorial disease caused by intricate interactions between multiple hereditary and environmental factors [2, 3]. Numerous studies have demonstrated that inflammation is critically implicated in the development of some cancers [4, 5]. In view of this, it is plausible that genetic polymorphisms in inflammation-related genes could modify cancer susceptibility [6–8].

Nuclear factor-kappa B (NF-κB) is a pleiotropic transcription factor discovered by Sen and Baltimore in 1986 [9]. In mammals, the NF-κB family consists of five members: c-Rel (Rel), Rel B, p65 (RelA), p50/p105 (NF-κB1), and p52/p100 (NF-κB2) [10]. This group of molecules function as key regulators of a variety of genes implicated in diverse biological events including cell survival, apoptosis, inflammation, differentiation, and autophagy [11, 12]. Recently, high levels of NF-κB have been observed in many cancers, including pancreatic cancer [13], lung cancer [14], colorectal cancer [15], breast cancer [16], melanoma [17], and multiple myeloma [18]. Although various dimeric forms of NF-κB exist, the most common is the p50 and p65/RelA heterodimer, encoded by the *NFKB1* and *RelA* genes, respectively [19].

The human *NFKB1* gene, spanning 156-kb, is located on chromosome 4q23-q24 and encodes a 105 kD protein (p105) which is cleaved into an active subunit (p50) [20]. Several variations have been identified in the *NFKB1* gene, including rs72696119 (C>G), rs28362491 (-94 ins/del ATTG), rs4648068 (A>G), and rs12509517 (G>C) [21]. Among these, *NFKB1* rs28362491, namely the -94insertion/deletion ATTG polymorphism, is potentially functional and the most widely investigated [21]. This modification occurs between two important regulatory elements (activator protein 1 and κB binding site) in the promoter region of the *NFKB1* gene. The deletion of four bases (ATTG) reduces or prevents the binding to nuclear proteins and leads to lower transcript levels of the *NFKB1* gene, thereby changing mRNA stability and regulating translation efficiency [21, 22].

Numerous case-control studies have assessed the association between the *NFKB1* -94ins/delATTG promoter polymorphism and cancer risk, with discrepant results. While some studies indicated an increased risk for some types of cancers [23–25], other studies showed instead a decreased risk, or no association [26, 27]. Several meta-analyses attempted to solve the controversy, but did not yield consistent results [28–33]. To provide a more precise evaluation of such association, we performed a comprehensive, updated meta-analysis. In addition, to minimize random errors and strengthen the robustness of our conclusions, we also performed trial sequential analysis (TSA).

## RESULTS

### Study characteristics

**Figure 1:**
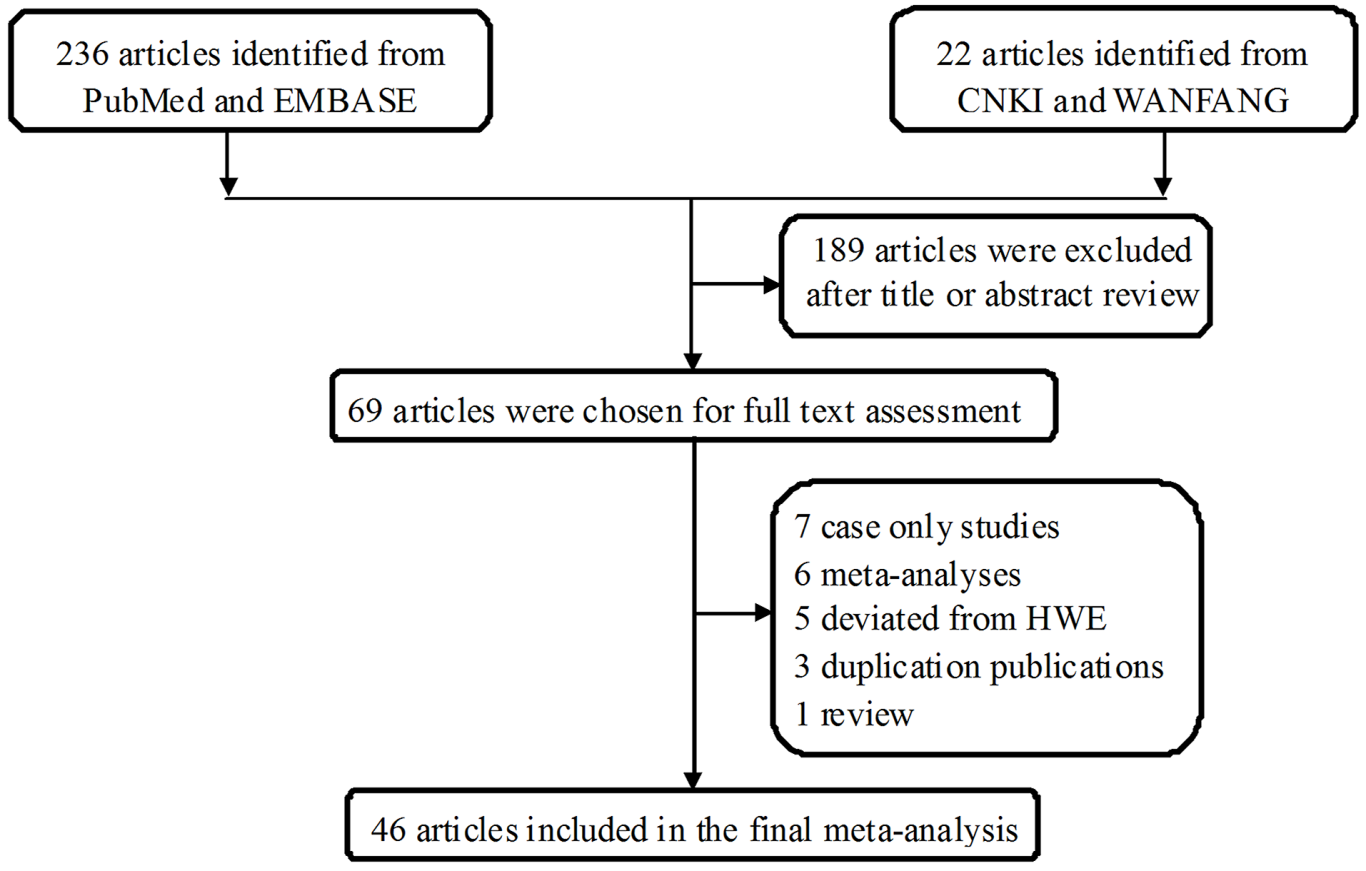
Flowchart of the study inclusion protocol

The study selection process for this meta-analysis is shown in Figure 1, 258 potentially relevant published records were retrieved from PubMed, EMBASE, China National Knowledge infrastructure (CNKI), and WANFANG databases. After screening the titles and reading the abstracts, 69 studies remained and were carefully reviewed. Among these, 23 publications were further excluded: 7 were case-only studies [34–39]; 6 were meta-analyses [28–33]; 5 deviated from Hardy-Weinberg equilibrium (HWE) [40–44]; 3 were duplicated publications [45–47]; 1 was a review [48], and 1 lacked sufficient data to calculate odds ratio (OR) and 95% confidence interval (CI) [49]. Thus, 46 publications were included in the final analysis [23–27, 48, 50–89]. Among these, publications that contained different case groups but used the same controls, or that studied one cancer type in different populations, were considered separate studies.

After this selection procedure, 50 studies extracted from 46 publications with 18,299 cases and 23,484 controls ultimately entered our final meta-analysis (Table 1). 38 of these studies included Asians subjects, and 12 included Caucasians. Regarding cancer types, 6 studies addressed hepatocellular carcinoma, 5 lung cancer, 4 colorectal cancer, 4 nasopharyngeal carcinoma, 4 prostate cancer, 4 ovarian cancer, 3 bladder cancer, 3 gastric cancer, 3 cervical cancer, 2 oral squamous cell carcinoma, 2 breast cancer, and 10 studies addressed other cancers. Moreover, 40 studies used a population-based design, and 10 were hospital-based. 19 studies had a quality score >9, and the remaining 31 had a quality score ≤9.

**Table 1:** Characteristics of the studies included in the current meta-analysis

Surname	Year	Cancer type	Country	Ethnicity	Control Source	Genotype method	Case		Control						MAF	HWE	Score
							II	ID	DD	All	II	ID	DD	All			
Lin [25]	2006	OSCC	China	Asian	HB	PCR-PAGE	59	103	50	212	43	100	58	201	0.54	0.993	7
Riemann [26]	2006	Colorectal cancer	Germany	Caucasian	HB	Pyro sequencing	54	58	27	139	118	141	48	307	0.39	0.586	9
Riemann [26]	2006	CLL	Germany	Caucasian	HB	Pyro sequencing	18	41	13	72	118	141	48	307	0.39	0.586	9
Riemann [26]	2006	RCC	Germany	Caucasian	HB	Pyro sequencing	47	76	17	140	118	141	48	307	0.39	0.586	9
Bu [48]	2007	Melanoma	Sweden	Caucasian	HB	PCR-RFLP	67	84	34	185	116	255	67	438	0.44	0.000	10
Riemann [83]	2007	Bladder cancer	Germany	Caucasian	HB	Pyro sequencing	88	124	30	242	118	141	48	307	0.39	0.586	10
Lehnerdt [27]	2008	HNSCC	Germany	Caucasian	HB	Pyro sequencing	132	179	53	364	118	141	48	307	0.39	0.586	8
He [82]	2009	HCC	China	Asian	HB	PCR-RFLP	83	84	35	202	97	183	124	404	0.53	0.070	9
He [89]	2009	HCC	China	Asian	HB	PCR-RFLP	55	65	30	150	70	136	94	300	0.54	0.130	8
Lo [24]	2009	Gastric cancer	China	Asian	HB	PCR	62	89	31	182	20	62	34	116	0.56	0.361	7
Zhang [81]	2009	Prostate cancer	China	Asian	HB	PCR-PAGE	46	57	14	117	44	68	31	143	0.45	0.624	8
Zhou [80]	2009	NPC	China	Asian	HB	PCR-PAGE	74	67	22	163	71	90	42	203	0.43	0.177	7
Tang [79]	2010	Bladder cancer	China	Asian	HB	PCR–PAGE	89	92	26	207	74	108	46	228	0.44	0.565	10
Zhou [78]	2010	Cervical cancer	China	Asian	HB	PCR–PAGE	108	105	20	233	135	166	64	365	0.40	0.297	9
Fan [77]	2011	Ovarian cancer	China	Asian	HB	PCR-CE	78	84	17	179	76	103	44	223	0.43	0.396	8
Lin [76]	2012	OSCC	China	Asian	HB	TaqMan	116	246	100	462	81	271	168	520	0.58	0.099	9
Song [92]	2012	Colorectal cancer	China	Asian	HB	PCR-RFLP	363	500	138	1001	297	522	186	1005	0.44	0.102	14
Tang [87]	2012	HCC	China	Asian	HB	PCR-RFLP	52	84	14	150	57	82	11	150	0.35	0.011	7
Ungerback [75]	2012	Colorectal cancer	Sweden	Caucasian	HB	TaqMan	114	187	43	344	256	270	96	622	0.37	0.079	8
Vangsted [74]	2012	Multiple myeloma	Denmark	Caucasian	PB	TaqMan	110	163	55	328	655	778	253	1686	0.38	0.303	7
Arisawa [73]	2013	Gastric cancer	Japan	Asian	PB	PCR-SSCP	172	239	68	479	342	435	103	880	0.36	0.046	11
Cheng [23]	2013	HCC	China	Asian	HB	TaqMan	42	64	29	135	81	271	168	520	0.58	0.099	7
Huang [72]	2013	Lung cancer	China	Asian	PB	TaqMan	372	459	225	1056	355	491	210	1056	0.43	0.090	10
Huang [72]	2013	Lung cancer	China	Asian	PB	TaqMan	169	230	104	503	189	289	145	623	0.46	0.092	10
Huo [71]	2013	Ovarian cancer	China	Asian	HB	Mass ARRAY	83	82	22	187	71	103	47	221	0.45	0.399	7
Kopp [70]	2013	Prostate cancer	Denmark	Caucasian	PB	RT-PCR	128	152	54	334	109	161	64	334	0.43	0.741	11
Li [69]	2013	Bladder cancer	China	Asian	HB	TaqMan	189	269	151	609	223	324	93	640	0.40	0.156	11
Liu [86]	2013	NPC	China	Asian	PB	TaqMan	116	135	49	300	86	143	71	300	0.48	0.443	12
Song [84]	2013	EC	China	Asian	HB	PCR-RFLP	42	52	6	100	56	39	5	100	0.25	0.588	6
Song [85]	2013	Cervical cancer	China	Asian	HB	PCR-RFLP	34	56	10	100	37	55	8	100	0.36	0.044	5
Umar [68]	2013	ESCC	India	Asian	HB	PCR	131	132	27	290	160	129	22	311	0.28	0.561	10
Gao [67]	2014	HCC	China	Asian	PB	TaqMan	68	102	40	210	171	160	79	410	0.39	0.000	12
Hua [66]	2014	Gastric cancer	China	Asian	HB	Mass ARRAY	92	182	127	401	120	230	83	433	0.46	0.144	9
Oltulu [65]	2014	Lung cancer	Turkey	Caucasian	HB	PCR-RFLP	35	44	16	95	46	47	6	99	0.30	0.194	7
Wang [64]	2014	Breast cancer	China	Asian	HB	PCR-RFLP	93	210	171	474	162	216	123	501	0.46	0.003	9
Zhang [63]	2014	HCC	China	Asian	PB	PCR	205	312	107	624	542	790	274	1606	0.42	0.631	10
Chen [62]	2015	Ovarian cancer	China	Asian	HB	Mass ARRAY	120	195	95	410	85	235	122	442	0.54	0.136	9
Cui [61]	2015	Prostate cancer	China	Asian	HB	PCR-RFLP	198	246	99	543	212	355	186	753	0.48	0.125	10
Han [60]	2015	Prostate cancer	China	Asian	PB	PCR-RFLP	63	339	534	936	38	331	567	936	0.78	0.230	12
Kopp [59]	2015	Colorectal cancer	Denmark	Caucasian	PB	KASP	320	449	146	915	679	787	253	1719	0.38	0.311	11
Li [58]	2015	Osteosarcoma	China	Asian	HB	PCR-RFLP	60	114	46	220	50	106	66	222	0.54	0.551	9
Liu [57]	2015	NPC	China	Asian	HB	TaqMan	236	331	117	684	274	438	195	907	0.46	0.420	9
Liu [57]	2015	NPC	China	Asian	HB	TaqMan	316	438	152	906	336	512	224	1072	0.45	0.262	9
Pallavi [56]	2015	Cervical cancer	Iran	Asian	HB	PCR-RFLP	98	116	26	240	73	104	113	290	0.57	0.000	9
Wang [55]	2015	Lung cancer	China	Asian	HB	PCR-RFLP	113	219	89	421	89	205	131	425	0.55	0.595	10
Wang [54]	2015	Thyroid carcinoma	China	Asian	HB	PCR-PAGE	106	186	60	352	171	209	79	459	0.40	0.273	11
Zhang [53]	2015	Lung cancer	China	Asian	HB	PCR-RFLP	434	252	32	718	352	290	76	718	0.31	0.162	9
Eskandari [52]	2016	Breast cancer	Iran	Asian	HB	AS-PCR	96	122	18	236	62	106	35	203	0.43	0.368	8
Lu [51]	2016	Ovarian cancer	China	Asian	HB	PCR-RFLP	115	351	221	687	95	339	253	687	0.61	0.271	10
Rybka [50]	2016	AML	Poland	Caucasian	HB	PCR	25	30	7	62	43	69	14	126	0.38	0.079	4

MAF, minor allele frequency; HWE, Hardy-Weinberg equilibrium; OSCC, oral squamous cell carcinoma; CLL, chronic lymphocytic leukemia; RCC, renal cell carcinoma; HNSCC, head and neck squamous cell carcinoma; HCC, hepatocellular carcinoma; NPC, nasopharyngeal carcinoma; ESCC, esophageal squamous cell carcinoma; EC, endometrial carcinoma; AML, acute myeloid leukemia; PB, population based; HB, hospital based; PCR-PAGE, polymerase chain reaction-polyacrylamide gel electrophoresis; PCR-RFLP, polymerase chain reaction-restriction fragment length polymorphism; PCR-CE, polymerase chain reaction-capillary electrophoresis; PCR-SSCP, polymerase chain reaction-single strand conformation polymorphism; KASP, kompetitive allele specific PCR; AS-PCR, allele-specific polymerase chain reaction.

### Meta-analysis results

**Figure 2:**
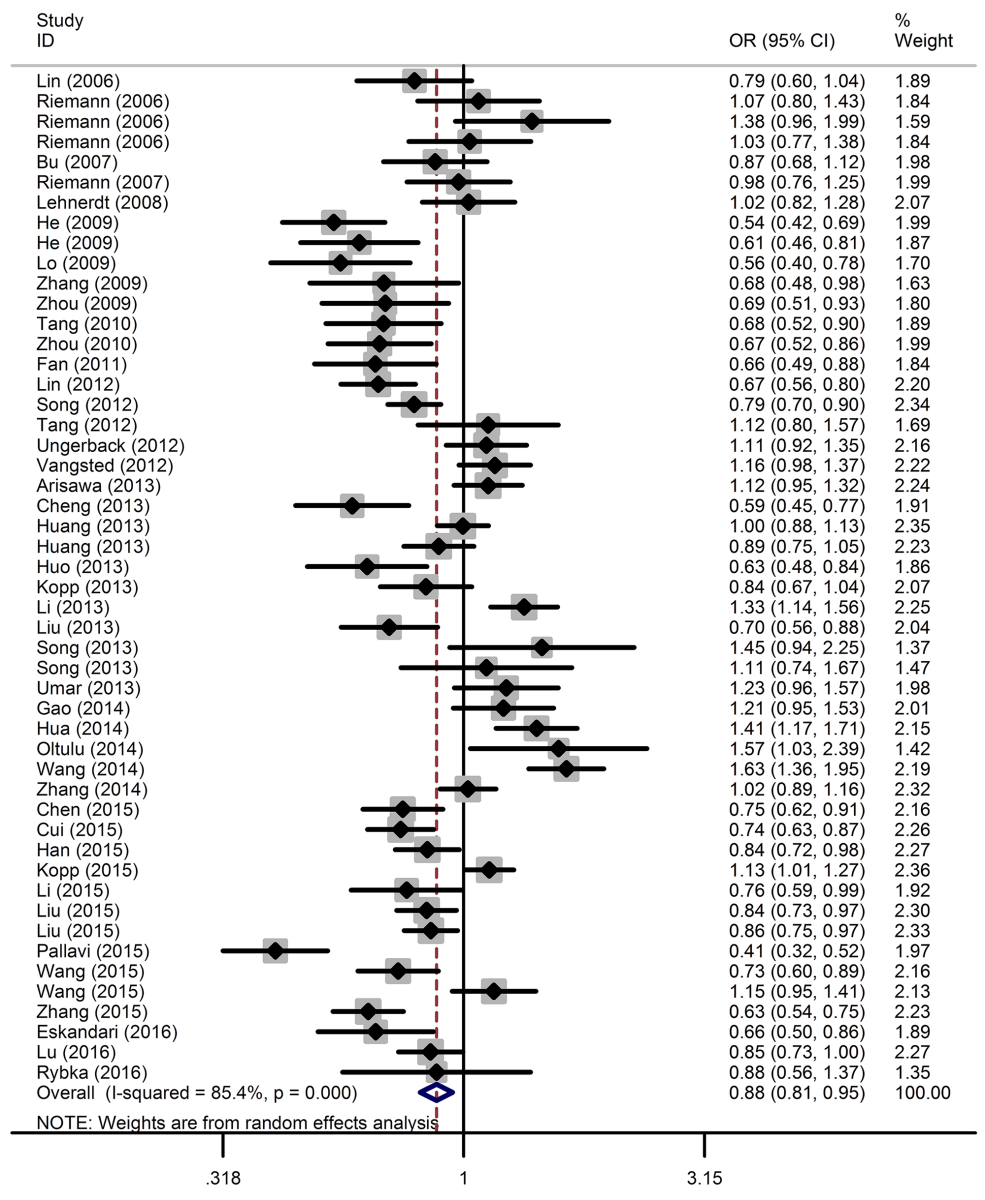
Forest plot of the association between the *NFKB1* -94ins/delATTG polymorphism and overall cancer susceptibility in the allele contrast model The horizontal lines represent the study-specific ORs and 95% CIs, respectively. The diamond represents the pooled OR and corresponding 95% CI.

The main results of this meta-analysis are shown in Table 2 and Figure 2. Overall, the pooled analysis demonstrated a significant, negative association between the *NFKB1* -94ins/delATTG polymorphism and overall cancer risk under all five genetic models (described in the Materials and Methods section): DD vs. II: OR = 0.75, 95% CI = 0.64-0.87; ID vs. II: OR = 0.91, 95% CI = 0.83-0.99; DD vs. ID/II: OR = 0.81, 95% CI = 0.71-0.91; ID/DD vs. II: OR = 0.86, 95% CI = 0.78-0.95; and D vs. I: OR = 0.88, 95% CI = 0.81-0.95.

**Table 2:** Meta-analysis of the association between the *NFKB1* -94ins/delATTG (rs28362491) polymorphism and overall cancer risk

Variables	No. of studies	Sample size	Homozygous	Heterozygous		Recessive		Dominant		Allele		
			DD vs. II	ID vs. II		DD vs. ID/II		ID/DD vs. II		D vs. I		
			OR (95% CI)	*P*^het^	OR (95% CI)	*P*^het^	OR (95% CI)	*P*^het^	OR (95% CI)	*P*^het^	OR (95% CI)	*P*^het^
All	50	18299/ 23484	**0.75 (0.64-0.87)**	<0.001	**0.91 (0.83-0.99)**	<0.001	**0.81 (0.71-0.91)**	<0.001	**0.86 (0.78-0.95)**	<0.001	**0.88 (0.81-0.95)**	<0.001
Cancer type												
HCC	6	1471/ 3390	0.65 (0.38-1.11)	<0.001	0.82 (0.56-1.19)	<0.001	0.74 (0.54-1.02)	0.006	0.75 (0.50-1.15)	<0.001	0.80 (0.61-1.07)	<0.001
Lung	5	2793/ 2921	0.77 (0.48-1.26)	<0.001	**0.84 (0.74-0.96)**	0.337	0.82 (0.54-1.26)	<0.001	0.83 (0.67-1.03)	0.012	0.88 (0.70-1.09)	<0.001
Colorectal	4	2399/ 3653	0.96 (0.65-1.43)	0.001	1.08 (0.79-1.47)	<0.001	0.92 (0.69-1.22)	0.022	1.05 (0.77-1.44)	<0.001	1.01 (0.82-1.24)	<0.001
NPC	4	2053/ 2482	**0.66 (0.56-0.78)**	0.467	**0.85 (0.75-0.97)**	0.537	**0.73 (0.63-0.85)**	0.755	**0.79 (0.69-0.90)**	0.371	**0.81 (0.74-0.89)**	0.316
Prostate	4	1930/ 2166	**0.59 (0.48-0.72)**	0.684	**0.74 (0.62-0.88)**	0.803	**0.77 (0.65-0.92)**	0.267	**0.69 (0.59-0.81)**	0.760	**0.79 (0.72-0.87)**	0.540
Ovarian	4	1463/ 1573	**0.54 (0.40-0.73)**	0.173	**0.73 (0.61-0.87)**	0.416	**0.68 (0.52-0.89)**	0.102	**0.67 (0.56-0.79)**	0.462	**0.75 (0.65-0.86)**	0.181
Bladder	3	1058/ 1175	0.93 (0.40-2.21)	<0.001	0.95 (0.74-1.22)	0.193	0.97 (0.43-2.17)	<0.001	0.96 (0.67-1.37)	0.026	0.97 (0.66-1.43)	<0.001
Gastric	3	1062/ 1429	0.97 (0.41-2.31)	<0.001	0.88 (0.60-1.30)	0.032	1.11 (0.57-2.15)	<0.001	0.90 (0.54-1.49)	0.002	0.98 (0.65-1.50)	<0.001
Cervical	3	573/ 755	0.41 (0.15-1.11)	0.001	0.85 (0.67-1.08)	0.627	0.44 (0.17-1.12)	0.001	0.69 (0.45-1.04)	0.050	0.66 (0.39-1.10)	<0.001
OSCC	2	674/ 721	**0.49 (0.33-0.72)**	0.222	**0.67 (0.51-0.88)**	0.570	**0.63 (0.49-0.81)**	0.308	**0.60 (0.46-0.77)**	0.377	**0.70 (0.60-0.82)**	0.300
Breast	2	710/ 704	0.92 (0.13-6.42)	<0.001	1.13 (0.51-2.54)	0.002	0.85 (0.20-3.61)	<0.001	1.13 (0.38-3.37)	<0.001	1.04 (0.43-2.53)	<0.001
Others	10	2113/ 4263	1.07 (0.88-1.31)	0.281	1.16 (0.94-1.42)	0.006	1.00 (0.86-1.16)	0.483	1.14 (0.94-1.38)	0.008	1.06 (0.95-1.19)	0.062
Ethnicity												
Asians	38	15079/ 18673	**0.67 (0.55-0.80)**	<0.001	**0.86 (0.79-0.94)**	<0.001	**0.75 (0.65-0.86)**	<0.001	**0.80 (0.72-0.89)**	<0.001	**0.83 (0.76-0.91)**	<0.001
Chinese	34	13834/ 16989	**0.68 (0.56-0.81)**	<0.001	**0.84 (0.77-0.93)**	<0.001	**0.77 (0.67-0.88)**	<0.001	**0.80 (0.71-0.89)**	<0.001	**0.84 (0.76-0.91)**	<0.001
Caucasians	12	3220/ 6559	1.08 (0.92-1.27)	0.221	1.11 (0.94-1.30)	0.004	1.02 (0.88-1.17)	0.269	1.10 (0.95-1.28)	0.010	1.06 (0.98-1.15)	0.141
Source of control												
HB	40	12614/ 15682	**0.70 (0.58-0.85)**	<0.001	**0.88 (0.80-0.98)**	<0.001	**0.76 (0.65-0.89)**	<0.001	**0.84 (0.74-0.94)**	<0.001	**0.85 (0.77-0.94)**	<0.001
PB	10	5685/ 9550	0.95 (0.79-1.15)	0.001	1.00 (0.86-1.15)	0.002	0.98 (0.88-1.09)	0.161	0.98 (0.84-1.14)	<0.001	0.98 (0.90-1.08)	<0.001
Quality score												
>9	19	9894/ 13117	0.87 (0.73-1.04)	<0.001	0.93 (0.84-1.04)	<0.001	0.92 (0.80-1.05)	<0.001	0.91 (0.81-1.03)	<0.001	0.94 (0.86-1.02)	<0.001
≤9	31	8405/ 12115	**0.68 (0.53-0.86)**	<0.001	0.89 (0.79-1.01)	<0.001	**0.73 (0.60-0.88)**	<0.001	**0.83 (0.71-0.96)**	<0.001	**0.84 (0.75-0.95)**	<0.001

Het, heterogeneity; HCC, hepatocellular carcinoma; NPC, nasopharyngeal carcinoma; OSCC, oral squamous cell carcinoma; HB, hospital-based; PB, population-based.

Stratified analysis by cancer type revealed that the -94ins/delATTG polymorphism significantly decreased lung cancer risk (ID vs. II: OR = 0.84, 95% CI = 0.74-0.96), nasopharyngeal carcinoma risk (DD vs. II: OR = 0.66, 95% CI = 0.56-0.78; ID vs. II: OR = 0.85, 95% CI = 0.75-0.97; DD vs. ID/II: OR = 0.73, 95% CI = 0.63-0.85; ID/DD vs. II: OR = 0.79, 95% CI = 0.69-0.90; D vs. I: OR = 0.81, 95% CI = 0.74-0.89), prostate cancer risk (DD vs. II: OR = 0.59, 95% CI = 0.48-0.72; ID vs. II: OR = 0.74, 95% CI = 0.62-0.88; DD vs. ID/II: OR = 0.77, 95% CI = 0.65-0.92; ID/DD vs. II: OR = 0.69, 95% CI = 0.59-0.81; D vs. I: OR = 0.79, 95% CI = 0.72-0.87), ovarian cancer risk (DD vs. II: OR = 0.54, 95% CI = 0.40-0.73; ID vs. II: OR = 0.73, 95% CI = 0.61-0.87; DD vs. ID/II: OR = 0.68, 95% CI = 0.52-0.89; ID/DD vs. II: OR = 0.67, 95% CI = 0.56-0.79; D vs. I: OR = 0.75, 95% CI = 0.65-0.86), and oral squamous cell carcinoma risk (DD vs. II: OR = 0.49, 95% CI = 0.33-0.72; ID vs. II: OR = 0.67, 95% CI = 0.51-0.88; DD vs. ID/II: OR = 0.63, 95% CI = 0.49-0.81; ID/DD vs. II: OR = 0.60, 95% CI = 0.46-0.77; D vs. I: OR = 0.70, 95% CI = 0.60-0.82). However, no correlation was observed between *NFKB1* -94ins/delATTG polymorphism and other types of cancer.

When stratified by population, a significant association between *NFKB1* -94ins/delATTG polymorphism and decreased cancer risk among Asians was detected under all genetic models (DD vs. II: OR = 0.67, 95% CI = 0.55-0.80; ID vs. II: OR = 0.86, 95% CI = 0.79-0.94; DD vs. ID/II: OR = 0.75, 95% CI = 0.65-0.86; ID/DD vs. II: OR = 0.80, 95% CI = 0.72-0.89; D vs. I: OR = 0.83, 95% CI = 0.76-0.91). As most of the studies were performed on the Chinese population, we determined the association of *NFKB1* -94ins/delATTG polymorphism with cancer risk on Chinese subjects. In this case, the results also showed a protective role against cancer (DD vs. II: OR = 0.68, 95% CI = 0.56-0.81; ID vs. II: OR = 0.84, 95% CI = 0.77-0.93; DD vs. ID/II: OR = 0.77, 95% CI = 0.67-0.88; ID/DD vs. II: OR = 0.80, 95% CI = 0.71-0.89; D vs. I: OR = 0.84, 95% CI = 0.76-0.91). No association was observed, however, for Caucasians.

Upon stratification based on the sources of controls, the *NFKB1* -94ins/delATTG polymorphism had a protective role against cancer in hospital-based groups (DD vs. II: OR = 0.70, 95% CI = 0.58-0.85; ID vs. II: OR = 0.88, 95% CI = 0.80-0.98; DD vs. ID/II: OR = 0.76, 95% CI = 0.65-0.89; ID/DD vs. II: OR = 0.84, 95% CI = 0.74-0.94; D vs. I: OR = 0.85, 95% CI = 0.77-0.94).

After stratification by quality score, a significantly decreased cancer risk was observed for studies with quality scores ≤9 (DD vs. II: OR = 0.68, 95% CI = 0.53-0.86; DD vs. ID/II: OR = 0.73, 95% CI = 0.60-0.88; ID/DD vs. II: OR = 0.83, 95% CI = 0.71-0.96; D vs. I: OR = 0.84, 95% CI = 0.75-0.95).

### Heterogeneity and sensitivity analysis

Statistically significant between-study heterogeneity was found in the pooled analysis under the five genetic models (*P* < 0.001). Thus, the random-effect model was applied to calculate the ORs and 95% CIs. Sensitivity analysis using the leave-one-out cross-validation method was conducted to assess the impact of each single study on the overall risk estimates. The omission of each individual study did not have substantial influence on the risk estimates, supporting the credibility and reliability of this meta-analysis (data not shown).

### Publication bias

**Figure 3:**
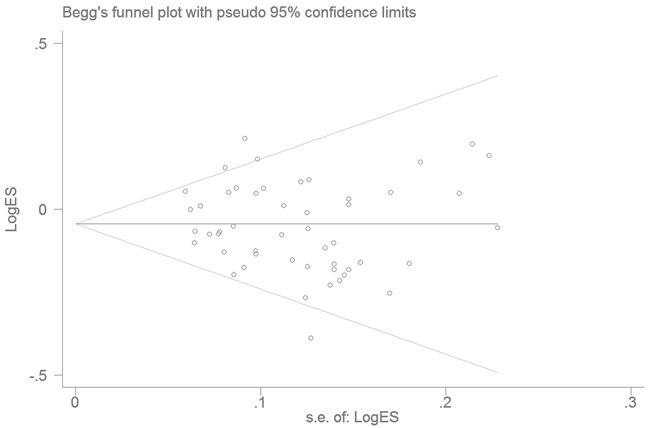
Funnel plot analysis to detect publication bias for *NFKB1* -94ins/delATTG polymorphism under the allele contrast model Each point represents a separate study.

Publication bias was assessed by Begg's funnel plot and quantitative Egger's test. The funnel plot showed a symmetrical shape (Figure 3), suggesting no publication bias, a conclusion further supported by the Egger's test (DD vs. II: *P* = 0.158; ID vs. II: *P* = 0.340, DD vs. ID/II: *P* = 0.157; ID/DD vs. II: *P* = 0.221; and D vs. I: *P* = 0.250).

### Trial sequential analysis and false-positive report probability (FPRP) analyses

**Figure 4:**
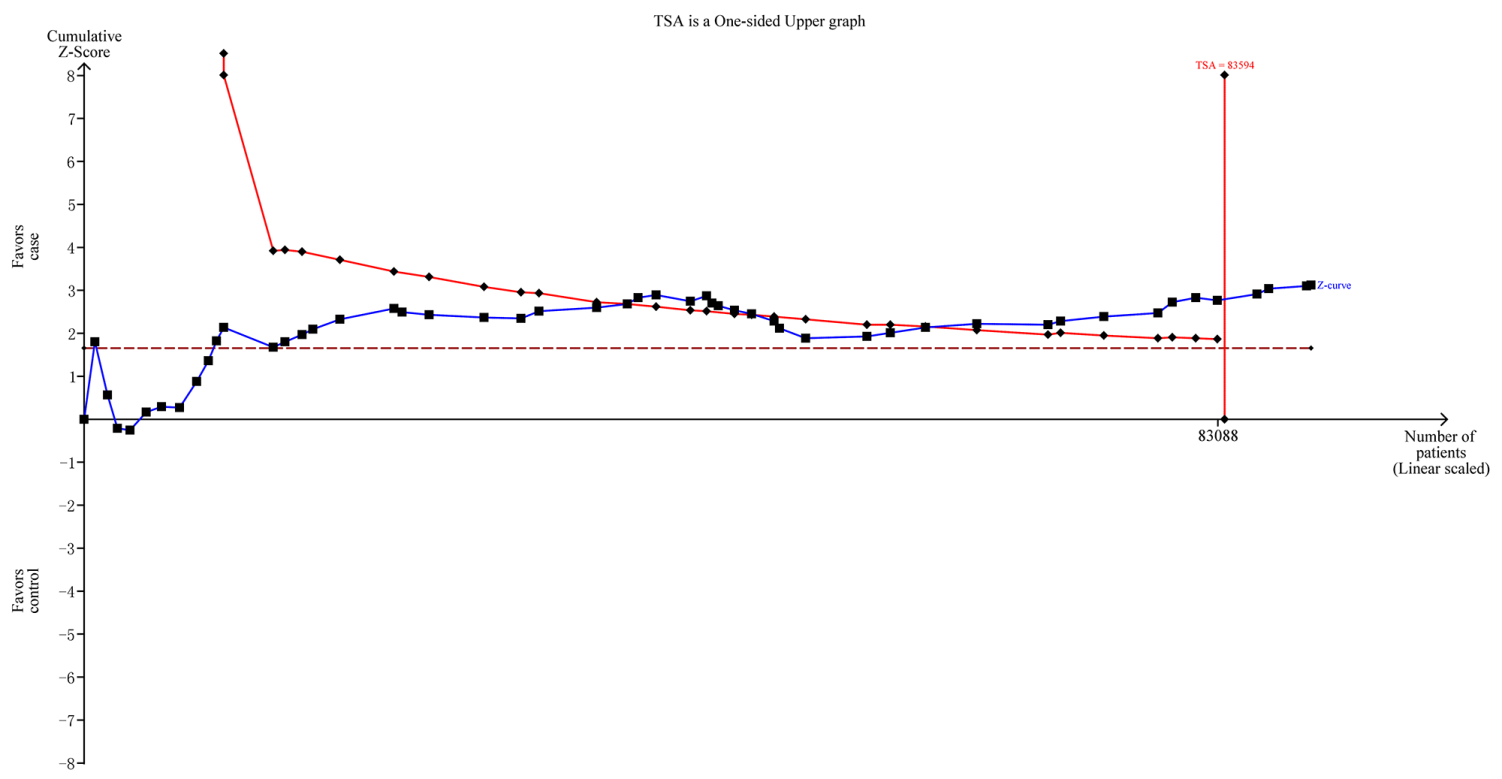
Trial sequential analysis for *NFKB1* -94ins/delATTG polymorphism under the allele contrast model

To minimize random errors and strengthen the robustness of our conclusions, we performed TSA (Figure 4). This analysis showed that the cumulative z-curve crossed the trial sequential monitoring boundary before reaching the required information size, suggesting that the cumulative evidence is sufficient and no further evidence is needed to verify the conclusions.

We finally calculated the FPRP values for all observed significant findings. With the assumption of a prior probability of 0.1, the FPRP values were all <0.20, suggesting that these significant associations were noteworthy (Table 3).

**Table 3:** False-positive report probability values for associations between the *NFKB1* -94ins/delATTG polymorphism and overall cancer risk

Variables	OR (95% CI)	*P* ^a^	Power ^b^	Prior Probability				
				0.25	0.1	0.01	0.001	0.0001
Homozygous (DD vs. II)								
All	0.75 (0.64-0.87)	2.45*10^-4^	1.000	0.001	0.002	0.024	0.197	0.710
NPC	0.66 (0.56-0.78)	1.99*10^-6^	0.652	0.000	0.000	0.000	0.003	0.030
Prostate	0.59 (0.48-0.72)	6.40*10^-7^	0.860	0.000	0.000	0.000	0.001	0.007
Ovarian	0.54 (0.40-0.73)	6.62*10^-6^	0.612	0.000	0.000	0.001	0.011	0.098
OSCC	0.49 (0.33-0.72)	3.26*10^-4^	0.388	0.003	0.008	0.077	0.457	0.894
Asians	0.67 (0.55-0.80)	1.81*10^-5^	1.000	0.000	0.000	0.002	0.018	0.153
Chinese	0.68 (0.56-0.81)	3.45*10^-5^	1.000	0.000	0.000	0.003	0.033	0.257
HB	0.70 (0.58-0.85)	3.30*10^-4^	1.000	0.001	0.003	0.032	0.248	0.767
QS ≤9	0.68 (0.53-0.86)	1.63*10^-3^	1.000	0.005	0.014	0.139	0.620	0.942
Heterozygous (ID vs. II)								
All	0.91 (0.83-0.99)	0.024	1.000	0.066	0.175	0.700	0.959	0.996
Lung	0.84 (0.74-0.96)	7.74*10^-3^	1.000	0.023	0.065	0.434	0.885	0.987
NPC	0.85 (0.75-0.97)	0.017	1.000	0.048	0.131	0.623	0.943	0.994
Prostate	0.74 (0.62-0.88)	7.44*10^-4^	0.998	0.002	0.007	0.069	0.427	0.882
Ovarian	0.73 (0.61-0.87)	5.68*10^-4^	0.979	0.002	0.005	0.054	0.367	0.853
OSCC	0.67 (0.51-0.88)	3.99*10^-3^	0.800	0.015	0.043	0.331	0.833	0.980
Asians	0.86 (0.79-0.94)	7.59*10^-4^	1.000	0.002	0.007	0.070	0.431	0.884
Chinese	0.84 (0.77-0.93)	4.59*10^-4^	1.000	0.001	0.004	0.043	0.314	0.821
HB	0.88 (0.80-0.98)	0.015	1.000	0.043	0.118	0.596	0.937	0.993
Recessive (DD vs. ID/II)								
All	0.81 (0.71-0.91)	3.22*10^-4^	1.000	0.001	0.003	0.031	0.243	0.763
NPC	0.73 (0.63-0.85)	3.26*10^-5^	0.841	0.000	0.000	0.004	0.037	0.279
Prostate	0.77 (0.65-0.92)	3.78*10^-3^	0.999	0.011	0.033	0.272	0.791	0.974
Ovarian	0.68 (0.52-0.89)	4.62*10^-3^	0.976	0.014	0.041	0.319	0.826	0.979
OSCC	0.63 (0.49-0.81)	2.37*10^-4^	0.356	0.002	0.006	0.062	0.399	0.869
Asians	0.75 (0.65-0.86)	6.02*10^-5^	1.000	0.000	0.001	0.006	0.057	0.376
Chinese	0.77 (0.67-0.88)	1.44*10^-4^	1.000	0.000	0.001	0.014	0.126	0.591
HB	0.76 (0.65-0.89)	5.23*10^-4^	1.000	0.002	0.005	0.049	0.343	0.840
QS ≤9	0.73 (0.60-0.88)	1.23*10^-3^	1.000	0.004	0.011	0.109	0.551	0.925
Dominant (ID/DD vs. II)								
All	0.86 (0.78-0.95)	2.68*10^-4^	1.000	0.008	0.024	0.210	0.728	0.964
NPC	0.79 (0.69-0.90)	2.94*10^-4^	0.997	0.001	0.003	0.028	0.227	0.747
Prostate	0.69 (0.59-0.81)	8.36*10^-6^	0.796	0.000	0.000	0.001	0.010	0.095
Ovarian	0.67 (0.56-0.79)	3.45*10^-6^	0.531	0.000	0.000	0.001	0.006	0.061
OSCC	0.60 (0.46-0.77)	9.87*10^-5^	0.186	0.002	0.005	0.050	0.347	0.842
Asians	0.80 (0.72-0.89)	9.05*10^-5^	1.000	0.000	0.001	0.009	0.083	0.475
Chinese	0.80 (0.71-0.89)	9.39*10^-5^	1.000	0.000	0.001	0.009	0.086	0.484
HB	0.84 (0.74-0.94)	2.29*10^-3^	1.000	0.007	0.020	0.185	0.696	0.958
QS ≤9	0.83 (0.71-0.96)	0.013	1.000	0.038	0.105	0.563	0.929	0.992
Allele (D vs. I)								
All	0.88 (0.81-0.95)	8.86*10^-4^	1.000	0.003	0.008	0.081	0.469	0.899
NPC	0.81 (0.74-0.89)	9.60*10^-6^	1.000	0.000	0.000	0.001	0.009	0.088
Prostate	0.79 (0.72-0.87)	1.03*10^-6^	1.000	0.000	0.000	0.000	0.001	0.010
Ovarian	0.75 (0.65-0.86)	3.47*10^-5^	1.000	0.000	0.000	0.003	0.033	0.257
OSCC	0.70 (0.60-0.82)	1.11*10^-5^	0.809	0.000	0.000	0.001	0.014	0.121
Asians	0.83 (0.76-0.91)	4.78*10^-5^	1.000	0.000	0.000	0.005	0.046	0.323
Chinese	0.84 (0.76-0.91)	9.24*10^-5^	1.000	0.000	0.001	0.009	0.085	0.480
HB	0.85 (0.77-0.94)	9.63*10^-4^	1.000	0.003	0.009	0.087	0.490	0.906
QS ≤9	0.84 (0.75-0.95)	4.84*10^-3^	1.000	0.014	0.042	0.324	0.829	0.980

CI, confidence interval; OR, odds ratio; NPC, Nasopharyngeal carcinoma; OSCC, oral squamous cell carcinoma; HB, Hospital based; QS, quality score.

^a^ Chi-square test was adopted to calculate the genotype frequency distributions.

^b^ Statistical power was calculated using the number of observations in the subgroup and the OR and *P* values in this table.

## DISCUSSION

In this meta-analysis, we found that the *NFKB1* -94ins/delATTG promoter polymorphism was significantly associated with decreased overall cancer risk under the five genetic models. To the best of our knowledge, this is the most comprehensive meta-analysis on this topic by now.

Numerous studies have suggested that polymorphisms in genes encoding inflammatory response factors, such as *TNF-alpha* -308G>A [6], *IL6* -174G>C [90], and *NFKBIA* -826C>T [91] may contribute to cancer susceptibility. Song et al. [92] reported that the *NFKB1* -94ins/delATTG polymorphism analyzed here (rs28362491) increased the risk of colorectal cancer in a Southern Chinese population; this association was also observed in several publications [71]. However, contradictory conclusions were also reported, namely a null association, or decreased cancer susceptibility. To address this controversy, at least six meta-analyses were performed. The first one, performed in 2011 by Zou et al. [32], included only 2,743 cases and 2,195 controls from 11 studies. They did not observe any association between the -94ins/delATTG variant and overall cancer. However, an ethno-specific association was detected by subgroup analysis; the D allele was protective against cancer in Asians, but increased the risk in Caucasians. Afterwards, Wang et al. [33] conducted an updated meta-analysis including 5,196 cases and 6,614 controls from 19 publications. They found that variant homozygotes (DD) had a decreased risk of cancer compared with wild-type homozygotes (II). The association was also found under the dominant genetic model (DD+DI vs. II). In subgroup analysis, a significantly decreased risk was observed in Asians but not in Caucasians. In addition, this susceptibility was cancer-specific, as it was observed for all cancer types examined, except for colorectal cancer. In 2014, four updated meta-analyses were published. Upon revision of 6,494 cases and 9,884 controls from 23 studies, Xu et al. [29] found that the -94ins/delATTG polymorphism was significantly associated with increased cancer risk under all the inheritance models. Stratified analysis by cancer type showed significant associations for ovarian cancer, hepatocellular carcinoma, and oral squamous cell carcinoma, but not for bladder cancer or lung cancer. Ethnicity subgroup analysis indicated that the polymorphism contributed to cancer risk in the Asian, but not the Caucasian, population. Another study by Yang et al. [28], which included 21 reports with 6,127 cases and 9,238 controls, also detected an increased overall cancer risk. Stratified analysis revealed a significant association between the polymorphism and ovarian, oral, and prostate cancers. These findings were also specific to the Asian population. Duan et al. [31] reviewed a total of 25 studies that included 8,750 cancer cases and 9,170 controls. They found that the insertion allele of the -94ins/delATTG polymorphism significantly increased cancer risk, both in overall genetic analysis as well as in Asians. Stratified analysis revealed that the polymorphism was associated with increased risk for oral squamous cell carcinoma and ovarian cancer, but not for colorectal cancer, bladder cancer, or renal cell cancer. In another meta-analysis involving 7,281 cases and 10,039 controls from 25 case-control studies, Nian et al. [30] found that the -94ins/delATTG polymorphism was significantly associated with decreased susceptibility to cancer in overall population under homozygous, recessive, dominant, and allele contrast models. Subgroups analysis based on ethnicity revealed that the polymorphism conferred decreased cancer susceptibility in the Asian population.

Since then, approximately 20 new relevant case-control studies in English and Chinese have emerged, some containing large samples and convincing results. Our study re-evaluated the impact of the *NFKB1* -94ins/delATTG polymorphism on cancer risk. In line with some previous meta-analyses, our pooled analysis revealed a significant association with decreased cancer risk under all five genetic models. Conversely, we found that the del allele of the *NFKB1* -94ins/delATTG polymorphism conferred a significantly decreased risk of cancer in the pooled analysis. Compared with the ins allele, the ins allele significantly enhances the binding ability to nuclear proteins and increases transcriptional activity, which eventually upregulates p50 (the active NF-κB1 subunit) expression [21]. Given the tumor-promoting role of p50 and NF-κB, it is biologically plausible that the -94del allele confers decreased cancer susceptibility.

In line with previous data, our study detected a significant association between the -94ins/delATTG polymorphism and cancer risk in Asians, but not in Caucasians, under all five genetic models. It is thus likely that the allelic distribution of this polymorphism vary geographically and ethnically, thus leading to the discrepancies in cancer risk. This may indicate that these groups have distinct environmental or genetic cancer co-etiologies. Stratification by cancer type showed that the *NFKB1* -94ins/delATTG polymorphism was inversely associated with the risk of lung cancer, nasopharyngeal carcinoma, prostate cancer, ovarian cancer, and oral squamous cell carcinoma, but no association was found for hepatocellular carcinoma, colorectal cancer, bladder cancer, gastric cancer, cervical cancer, breast cancer, or other cancers. This phenomenon may be partly attributed to the inherent heterogeneity of oncogenic progression in different cancer types [93], although the insufficient statistical power caused by the relatively small number of studies on each cancer type may also be a factor.

The credibility of our conclusions is supported by the inclusion of Chinese-language studies, exclusion of publications with controls violating the Hardy-Weinberg equilibrium, and inclusion of subgroup, publication bias, and sensitivity analyses. Among the limitations of our meta-analysis are a significant between-study heterogeneity, detected in some comparisons, which may diminish the strength of our conclusions. The source of this heterogeneity may be ascribed to sample size, genotyping methods, ethnicity, source of controls, as well as the studies’ diverse quality scores. Second, we assessed the association between the *NFKB1* -94ins/delATTG polymorphism and cancer risk from a genetic perspective only, by using unadjusted ORs. Multiple potentially influential factors such as life style, environmental exposure, and gene-environment interactions should be considered to obtain a more precise risk estimation. Third, the number of studies in certain subgroup analyses was too small to obtain a reliable association. For instance, only six publications were included for hepatocellular carcinoma, and fewer studies were available for breast cancer and oral squamous cell carcinoma, which restrains further analysis for risk factors. Finally, the meta-analysis is a type of retrospective study with several inherent drawbacks: inconsistent qualities of primary studies, incomplete histological details, misclassified genotypes, different definitions of disease status, and improperly matched sources of controls.

In conclusion, despite these limitations, and in agreement with several previous studies, this meta-analysis draws the robust conclusion that *NFKB1* -94ins/delATTG polymorphism is associated with decreased cancer risk, especially in the Asian population. These findings indicate a possible involvement of *NFKB1* in the etiology of tumorigenesis, and suggest the potentially relevant therapeutic value of NF-κB modulation in cancer prevention. Further multi-center, well-designed investigations with larger sample sizes that include gene-environment interactions assessment are warranted to confirm our findings.

## MATERIALS AND METHODS

### Publication search

We performed a comprehensive literature search by using the PubMed and EMBASE databases, without language limitations, up to July 1, 2016. The following search terms were used: “polymorphism or SNP or single nucleotide polymorphism or variant” and “NFKB1/NF-κB1 or nuclear factor kappa B1”, and “tumor or cancer or neoplasm or carcinoma”. We also searched the China National Knowledge Infrastructure (CNKI) and WANFANG databases to obtain additional, relevant studies. Retrieved articles were manually screened to determine eligible studies. When two or more publications containing overlapping data were found, the largest study was included in the final meta-analysis.

### Inclusion/exclusion criteria

All articles included in the current analysis met the following criteria: 1) evaluation of the association between *NFKB1* -94ins/delATTG polymorphism and cancer risk; 2) case-control studies; 3) sufficient information provided to estimate ORs and 95% CIs; 4) *NFKB1* -94ins/delATTG genotype frequency in agreement with HWE in controls. Exclusion criteria were as follows: 1) case-only studies; 2) meta-analysis or reviews; 3) studies that lacked detailed genotyping data; 4) duplicates of previous publications.

### Data extraction

Two authors (Z.Z. and W.F.) evaluated all eligible studies independently and extracted the following information: first author's surname, year of publication, cancer type, country, ethnicity, source of controls, genotyping methods, and genetic distribution of cases and controls. Stratification analyses were conducted by cancer type, ethnicity (Asians, Caucasians), source of control (hospital-based and population-based) and quality score (>9 and ≤9). If a study contained two or more ethnic groups or cancer types, we divided the study accordingly.

### Trial sequential analysis

TSA was performed as described by us previously [94]. Briefly, after adopting a level of significance of 5% for type I error and of 30% for type II error, the required information size was calculated, and TSA monitoring boundaries were built.

### FPRP analysis

The FPRP values at different prior probability levels for all significant findings were calculated as described by us previously [95]. Briefly, 0.2 was set as FPRP threshold and assigned a prior probability of 0.01 to detect an OR of 0.67 (for protective effects) for an association with genotypes under investigation. A FPRP value <0.2 denoted a noteworthy association.

### Statistical methods

Goodness-of-fit χ^2^ test was used to assess HWE in the control subjects. Departure from HWE was assessed using a *P* < 0.05 as threshold in each study. The strength of the association between *NFKB1* -94ins/delATTG polymorphism and cancer risk was assessed by calculating ORs and corresponding 95% CIs. Five genetic models were adopted: homozygote model (DD, homozygous deletion (del/del) vs. II, homozygous insertion (ins/ins) or wild-type); heterozygote model (ID, heterozygous ins/del vs. II); recessive model (DD vs. ID/II); dominant model (ID/DD vs. II); and allele contrast model (D vs. I). Subgroup and stratification analyses were also performed to test the association by ethnicity, cancer type, source of control and quality score. We performed χ^2^-based Q-test to assess heterogeneity between study results. The fixed-effects model (Mantel-Haenszel method) was used if the studies were found to be homogeneous (with *P* > 0.10 for the Q-test). Otherwise, the random-effects model (DerSimonian and Laird method) was adopted to estimate the pooled OR [96–99]. Quality assessment for each study was performed using the quality assessment criteria described previously (Supplementary Table 1) [98]. Sensitivity analysis was carried out by individually removing each study and reanalyzing the pooled risk estimates. Potential publication bias was estimated by Begg's funnel plot and Egger's linear regression, where an asymmetric plot and a *P* value < 0.05, respectively, indicate the presence of publication bias. All the data management and statistical analyses were completed using STATA software (Stata Corporation, College Station, TX; version 11.0). All the *P* values were two-sided. A *P* value of < 0.05 was considered statistically significant.

## SUPPLEMENTARY TABLES


