# Supplementary material for: NFKB1-94insertion/deletion ATTG polymorphism and cancer risk: Evidence from 50 case-control studies
Source: Oncotarget. 2016 Dec 26;8(6):9806–22. doi: 10.18632/oncotarget.14190 (PMC5354772; doi:10.18632/oncotarget.14190)
Supplement: Supplementary file 1 [file oncotarget-08-9806-s001.pdf]

NFKB1 -94insertion/deletion ATTG polymorphism and cancer risk: Evidence from 50 case-control studies

SUPPLEMENTARY TABLES

Supplementary Table 1: Quality assessment scoring for the included studies.

Criteria	Score
Representativeness of cases	
Selected from population cancer registry	2
Selected from hospital	1
No method of selection described	0
Representativeness of controls	
Population-based	3
Blood donors	2
Hospital-based	1
Not described	0
Ascertainment of cancer cases	
Histopathologic confirmation	2
Patient medical record	1
Not described	0
Control selection	
Controls matched with cases by age and sex	2
Controls matched with cases only by age or by sex	1
Not matched or not described	0
Genotyping examination	
Genotyping done blindly and quality control	2
Only genotyping done blindly or quality control	1
Unblinded and without quality control	0
Total sample size for both cases and controls	
Larger than 1000	3
Larger than 500, but less than 1000	2
Larger than 200, but less than 500	1
Less than 200	0
